# Deficient Cholesterol Esterification in Plasma of *apoc2* Knockout Zebrafish and Familial Chylomicronemia Patients

**DOI:** 10.1371/journal.pone.0169939

**Published:** 2017-01-20

**Authors:** Chao Liu, Daniel Gaudet, Yury I. Miller

**Affiliations:** 1 Department of Medicine, University of California San Diego, La Jolla, California, United States of America; 2 Department of Medicine, ECOGENE-21 Clinical and Translational Research Center and Lipidology Unit, Université de Montréal, Chicoutimi, Quebec, Canada; University of Milano, ITALY

## Abstract

Hypertriglyceridemia is an independent risk factor for cardiovascular disease. Apolipoprotein C-II (APOC2) is an obligatory cofactor for lipoprotein lipase (LPL), the major enzyme catalyzing plasma triglyceride hydrolysis. We have created an *apoc2* knockout zebrafish model, which mimics the familial chylomicronemia syndrome (FCS) in human patients with a defect in the *APOC2* or *LPL* gene. In this study, we measured plasma levels of free cholesterol (FC) and cholesterol esters (CE) and found that *apoc2* mutant zebrafish have a significantly higher FC to CE ratio (FC/CE), when compared to the wild type. Feeding *apoc2* mutant zebrafish a low-fat diet reduced triglyceride levels but not the FC/CE ratio. In situ hybridization and qPCR results demonstrated that the hepatic expression of lecithin-cholesterol acyltransferase *(lcat)*, the enzyme responsible for esterifying plasma FC to CE, and of apolipoprotein A-I, a major protein component of HDL, were dramatically decreased in *apoc2* mutants. Furthermore, the FC/CE ratio was significantly increased in the whole plasma and in a chylomicron-depleted fraction of human FCS patients. The FCS plasma LCAT activity was significantly lower than that of healthy controls. In summary, this study, using a zebrafish model and human patient samples, reports for the first time the defect in plasma cholesterol esterification associated with LPL deficiency.

## Introduction

Hypertriglyceridemia is an independent risk factor for cardiovascular disease, and human genetic studies suggest that reduced triglyceride (TG) levels in the carriers of APOC3 and ANGPTL4 loss-of-function mutations correlate with the decreased risk of heart attack [1–4]. APOC3 and ANGTL4 are both inhibitors of lipoprotein lipase (LPL), which is the key enzyme responsible for plasma TG hydrolysis. In contrast, APOC2 is an obligatory co-activating factor for LPL [5]. The familial chylomicronemia syndrome (FCS) patients, who have deficiency in APOC2 or LPL, consuming normal diet develop severe hypertriglyceridemia and chylomicronemia and often manifest eruptive xanthomas, lipemia retinalis, and acute and recurrent pancreatitis [6–10]. Currently, there are no effective approved therapies for FCS patients, but recent clinical trials show promising results of an APOC3 antisense oligonucleotide therapy to dramatically reduce TG levels in FCS patients [11].

It is well documented that in FCS patients TG-rich lipoproteins, chylomicrons and VLDL, are dramatically increased but cholesterol-rich LDL and HDL are decreased [12, 13]. The decreased LDL-C levels are mainly due to the defective TG hydrolysis of VLDL, and the LDL in FCS patients is in fact more similar to VLDL in terms of the increased ratio of TG to cholesterol. Changes in the apolipoprotein composition of HDL, such as reduced apoA-I, are the likely cause of decreased HDL-C in FCS patients [12–14]. It is also possible that the prolonged action of cholesteryl ester transfer protein (CETP) on the VLDL with higher TG levels triggers excessive transfer of CE to VLDL and of TG to HDL, resulting in reduced HDL-C [15, 16]. Lecithin:cholesterol acyltransferase (LCAT) is another enzyme involved in lipoprotein remodeling in plasma. LCAT catalyzes the transfer of a fatty acid from phosphatidylcholine (lecithin) to unesterified (free) cholesterol (FC). The resulting cholesteryl esters (CE) are stored in the hydrophobic core of HDL to be transferred to the liver. In familial LCAT deficiency (FLD) patients, loss of function of LCAT results in lower plasma HDL-C, which may contribute to the pathogenesis of corneal opacity, dyslipidemia and proteinuria with a poor renal prognosis [17]. Overexpression of human *LCAT* in squirrel monkeys, a non-human primate model, increased HDL-C by 100%, and recombinant human LCAT increased the HDL-C in a phase 1 clinical trial [18–20]. However, LCAT regulation under the conditions of LPL or APOC2 deficiency was not studied.

Zebrafish is an emerging model to study lipid metabolism and vascular mechanisms relevant to the pathogenesis of human atherosclerosis. The genes involved in lipid and lipoprotein metabolism, such as *APOC2*, *LPL*, *LCAT* and *CETP*, are conserved from zebrafish to humans [21–24]. In our previous study, we have developed an *apoc2* knockout zebrafish model, which replicates many aspects of human FCS, including a pronounced hypertriglyceridemia, associated with an increase in chylomicrons and VLDL, and decreased LDL and HDL [25]. It is the first animal model with a total loss-of-function of *apoc2*. In the present study, we found a significant increase in the plasma ratio of FC to CE (FC/CE) in *apoc2* mutant zebrafish and in human FCS patients, which was associated with reduced *lcat* expression in zebrafish and reduced LCAT activity in human plasma.

## Materials and Methods

### Ethics statement

All animal experiments were performed according to the NIH guidelines and were approved by the University of California, San Diego Institutional Animal Care and Use Committee (protocol S07266). The collection of human blood samples from participants who provided written informed consent was approved by the Institutional Review Board of UC San Diego (project #71402) and by ECOGENE-21 Clinical and Translational Research Center IRB Services (project #00013642).

### Zebrafish maintenance and feeding

Adult zebrafish, wild type (AB strain) and *apoc2* mutants (on the AB background) [25], were maintained at 28°C, 14-h-light–10-h-dark cycle and fed live brine shrimp twice a day. The low fat diet (LFD) was prepared by extracting lipid from Zebrafish Select Diet (Aquaneering, San Diego, CA) with diethyl ether. Zebrafish were euthanized with overdose of tricaine methanesulfonate (MS-222, 250–300mg/L) by prolonged submersion. Zebrafish were be left in the solution for at least 10 minutes following cessation of opercular movement. This method of euthanasia is consistent with the recommendations of 2010 Report of the AVMA Panel on Euthanasia.

### Plasma triglyceride and cholesterol measurements

Blood was collected from adult male zebrafish after overnight fasting through tail amputation and diluted 1:50 (WT) or 1:200 (*apoc2* mutant) in PBS. The supernatants were collected as a plasma fraction after centrifugation at 2,350 g for 10 min. Human blood was collected from healthy volunteers or FCS patients according to the protocols approved by the Institutional Review Boards of University of California, San Diego and Université de Montréal. To deplete chylomicrons from FCS plasma, the whole plasma was centrifuged at 15,000 g for 10 min and the lower clear fraction was collected as a chylomicron-depleted plasma (FCS-CD). TG and cholesterol levels were measured using kits from BioVision (Milpitas, CA; Triglyceride Quantification Kit, K622-100; Cholesterol Quantification Kit, K623-100), according to the manufacturer’s protocols.

### RNA in situ hybridization and qPCR

Digoxigenin-labeled probes were synthesized using an *in vitro* transcription system (Roche). Whole-mount in situ hybridization was performed as described [26]. Images were captured with a Leica CTR5000 microscope (Wetzlar, Germany). For gene expression analyses, whole body homogenates of embryos at 5.3 days post fertilization (dpf) and homogenates of livers dissected from 8 month old male adult zebrafish were used. Total RNA was extracted with Trizol (Thermo Fisher, Cat#15596026) following the manufacturer’s protocol. cDNA was prepared with an RNA to cDNA EcoDry kit (Takara-Clontech, Cat#639543) and qPCR was performed with a SYBR fast qPCR kit (Kapa, Cat#KK4602), using a Rotor Gene Q thermocycler (Qiagen). The qPCR primers used in these studies were: *beta-actin* (GenBank, NM_131031), 5’GGCTTCTGCTCTGTATGG3’ and 5’AACGCTTCTGGAATGACTAA3’; *lcat* (GenBank, XM_001332792), 5’CGGTTACTTCCACACTATG3’ and 5’TACTCCTCCTGCTCATTC3’; *cetp* (GenBank, XM_009293552), 5’CCATAATGACGGACGATT3’ and 5’ATGACTCTGACTGATGTG3’; *apoa1* (GenBank, NM_131128), 5’GCACTGACTCTTCTCTTG3’ and 5’CTGATCCTTGACCTGGTT3’; *apoe* (GenBank, NM_131098), 5’CCTCTGATGCTGCTGGTC3’ and 5’CTGAGTGCTGCGTTCCTT3’; *apoB* (GenBank, XM_689735), 5’AGAGGCTTAGAGATATGCTGAGT3’ and 5’GGCGTGGATGTTGCTTGA3’; and *mtp* (GenBank, NM_212970), 5’GATAACGGCAAACTCTACA3’ and 5’GCTAATCCTGAATCCAACA3’.

### LCAT activity assay

The LCAT activity in human plasma was assessed using a protocol modified from the LCFC-LCAT Acyltransferase Activity Assay kit (Sigma/Roar Biomedical, Cat# MAK306), which measures consumption of free cholesterol in an LCAT reaction. In brief, undiluted plasma was incubated at 37°C for 60 min, in the absence and presence of an LCAT inhibitor (sodium iodoacetate, Sigma, Cat# 57858, at a final concentration of 20 mg/ml), and then placed on ice. At the end of incubation, the control tube was supplemented with an equal amount of sodium iodoacetate. Levels of free cholesterol were measured using a Cholesterol Quantification kit (Biovision, Cat# K623-100). LCAT activity was calculated as the difference in free cholesterol levels between control and LCAT-inhibited samples, and normalized to the mean of LCAT activity in healthy individuals.

### Statistical analyses

Graphs represent means ± standard error from 3–5 independent experiments. Results were analyzed using Student's t-test and the differences with p<0.05 were considered statistically significant.

## Results and Discussion

**Fig 1:**
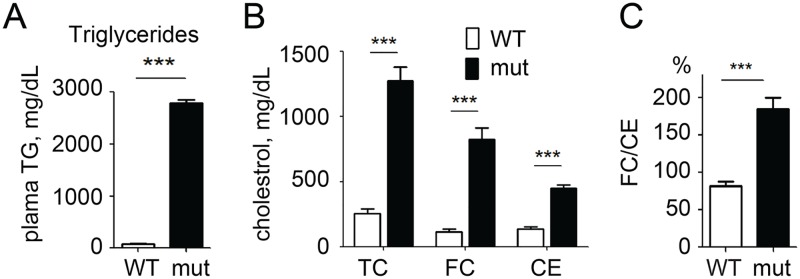
Disproportional free cholesterol (FC) and cholesterol ester (CE) levels in *apoc2* mutant zebrafish. (A) Plasma triglyceride (TG) levels in adult wild type (WT) and *apoc2* mutant zebrafish; (B) Plasma total cholesterol (TC), FC and CE levels; (C) FC/CE ratio. Results are mean±s.e.m.; n = 3 in each group; ***P<0.001 (Student’s t-test). Graphs in panel A and part of panel B replicate previously reported results [25].

**Fig 2:**
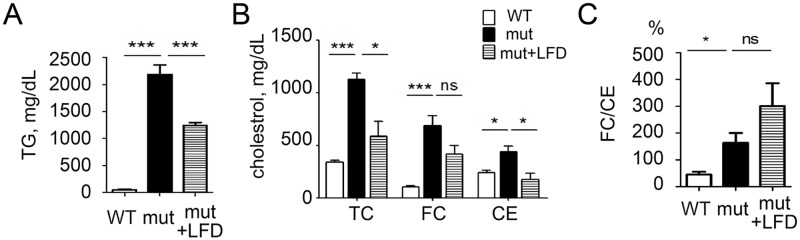
Reducing hypertriglyceridemia does not correct the FC/CE ratio. Plasma TG levels (A), cholesterol levels (B) and the FC/CE ratio (C) in WT, *apoc2* mutants and the *apoc2* mutants fed a low fat diet (LFD). Results are mean±s.e.m.; n = 3 in each group; *P<0.05, ***P<0.001 (Student’s t-test).

As we have previously reported [25], adult *apoc2* mutants have severe hypertriglyceridemia (Fig 1A) and pronounced hypercholesterolemia (Fig 1B). Interestingly, we found a disproportionate increase in the levels of FC, as compared to CE, and, accordingly, the FC/CE ratio was higher in *apoc2* mutants than in WT (Fig 1B and 1C). One explanation for this finding could be that a large surface area of triglyceride rich lipoproteins (TRLs) provides an extra space for amphiphilic cholesterol inserted in the phospholipid monolayer, while the core of TRLs is largely filled with TG but not CE. To test this possibility, we fed adult *apoc2* mutants an LFD for two weeks and measured plasma TG and cholesterol levels. As expected, plasma TG and TC levels were reduced in *apoc2* mutants fed with LFD (Fig 2A and 2B). However, the FC/CE ratio was not reduced and even trended higher in *apoc2* mutants fed an LFD (Fig 2B and 2C). This result suggests that the disproportionate FC and CE levels in *apoc2* mutants were independent of plasma TG levels.

**Fig 3:**
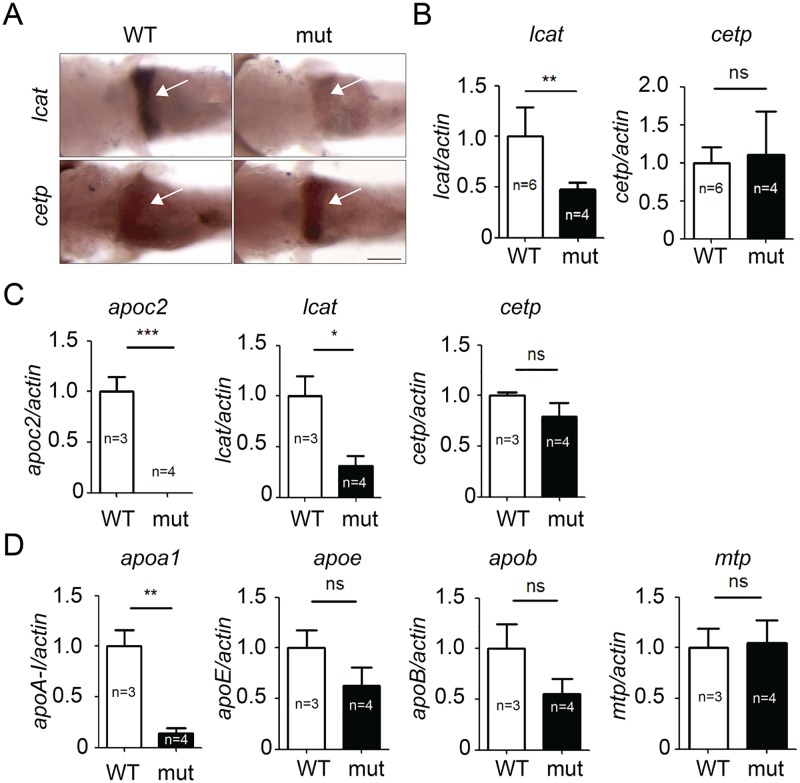
Expression of *lcat*, but not *cetp*, is significantly decreased in *apoc2* mutant zebrafish. In situ hybridization (A) and qPCR (B) results showing *lcat* and *cetp* mRNA expression in 5.3 dpf zebrafish embryos. mRNA expression of *apoc2*, *lcat* and *cetp* (C) and *apoa1*, *apoe*, *apob* and *mtp* (D) in adult zebrafish liver. Results are mean±s.e.m.; numbers of biological replicates are indicated on the graphs; *P<0.05, **P<0.01 and ***P<0.001 (Student’s t-test).

Next, we tested the hypothesis that the *apoc2* knockout leads to a defective plasma lipoprotein remodeling by Cetp and/or Lcat. As in mammals, zebrafish *lcat* and *cetp* are mainly expressed in the liver. In situ hybridization of 5.3 dpf zebrafish showed no changes in the liver *cetp* mRNA expression, but *lcat* was dramatically decreased in the liver of *apoc2* mutants (Fig 3A). This result was confirmed with RT-qPCR of RNA isolated from whole body homogenates of 5.3 dpf zebrafish (Fig 3B). We also performed RT-qPCR using homogenates of liver dissected from adult zebrafish, and the results were consistent with those in larvae—decreased *lcat* but no changes in *cetp* expression (Fig 3C). Furthermore, expression of *apoa1* was dramatically decreased in the liver of *apoc2* mutants, whereas changes in the expression of *apoe*, *apob* and *mtp* were not significant (Fig 3D). These results suggest that the disproportionately high FC levels in *apoc2* mutant zebrafish were likely due to a defect in Lcat-catalyzed cholesterol esterification and/or reduced apoA-I production by the liver.

**Fig 4:**
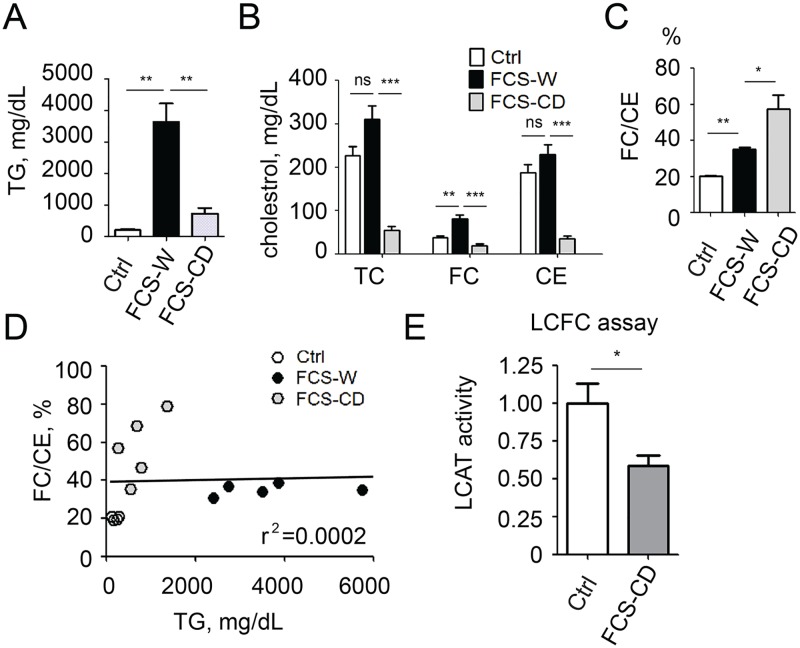
Patients with familial chylomicronemia syndrome (FCS) have disproportional FC and CE levels in plasma. TG levels (A), cholesterol levels (B) and the FC/CE ratio (C) in plasma of healthy subjects (Ctrl), in FCS patients’ whole plasma (FCS-W), and in chylomicron-depleted FCS plasma (FCS-CD). (D) A linear regression analysis of the FC/CE ratio and plasma TG levels (n = 4 in control group and n = 5 in FCS-W and FCS-CD groups). (E) LCAT activity in healthy subjects’ and FCS-CD plasma (n = 5 in control group and n = 6 in FCS-CD group). Results are mean±s.e.m.; *P<0.05, **P<0.01 and ***P<0.001 (Student’s t-test).

To test whether our findings in *apoc2* knockout zebrafish are relevant to human patients, we examined plasma from FCS patients with genetically validated LPL deficiency. The FCS plasma has a milky appearance due to high content of chylomicrons. In addition to testing whole plasma from FCS patients (FCS-W), we also made a chylomicron-depleted plasma (FCS-CD) by spinning FCS-W without adjusting density at 15,000 g and collecting the clear layer under the topper-most, white layer of chylomicrons. As expected, TG levels were substantially lower in FCS-CD compared to FCS-W (Fig 4A). Cholesterol levels were also decreased in FCS-CD compared to FCS-W (Fig 4B) and were even lower than in the control plasma, which is consistent with the reports that FCS patients have reduced LDL-C and HDL-C levels when compared to normal subjects [16]. In agreement with the zebrafish results, the FC/CE ratio was increased in FCS-W and, interestingly, was even higher in FCS-CD plasma (Fig 4C). Also consistent with the zebrafish results, there was no correlation between the FC/CE ratio and plasma TG levels in human plasma (Fig 4D).

Next, we measured LCAT activity and found that it was significantly lower in FCS-CD compared to control plasma (Fig 4E). These results suggest that a disproportionate increase in unesterified cholesterol in FCS patients’ plasma can be due to reduced LCAT activity, although we cannot exclude the possibility that this is due to the reduced FC substrate levels in FCS plasma. Furthermore, our data imply that a mechanism linking the LPL deficiency with a defect in LCAT-mediated cholesterol esterification can be conserved from zebrafish to humans.

A major source of FC in plasma is the unesterified cholesterol removed together with phospholipids from cells by lipid poor apoA-I to produce pre-beta HDL and by mature HDL. To maintain the HDL capacity to accept more FC, LCAT esterifies FC into CE, which due to its hydrophobicity moves into the hydrophobic core of HDL. Thus, the LCAT deficiency we identified in *apoc2* mutant zebrafish and in FCS patients’ plasma may limit HDL cholesterol efflux capacity, which in turn contributes to the risk of cardiovascular disease [27, 28]. This possibility can be evaluated in future studies.

The reason for LCAT deficiency in organisms in which LPL activity is inhibited, is unclear. Some studies suggest that apoA-I, which is an important factor in LCAT activation, is decreased in plasma of FCS patients [6, 13]. In agreement, we found that the hepatic *apoa1* mRNA expression was decreased in adult *apoc2* mutant zebrafish (Fig 3D). Thus, reduced apoA-I expression can be an important factor in the LCAT activity defect. However, we also found a significantly reduced *lcat* expression in the liver of *apoc2* mutant zebrafish (Fig 3). There is no FCS patient data available to corroborate the findings of reduced LCAT expression in zebrafish liver. It is unlikely that APOC2 has a direct effect on LCAT activity because patients with a primary LPL deficiency (but normal APOC2) had reduced LCAT activity in our study. We speculate that the loss of LPL/APOC2-mediated TG lipolysis may affect liver expression of LCAT and/or apoA-I and thus result in defective HDL metabolism. Previous studies have reported that hypertriglyceridemic patients (including FCS patients) have reduced HDL-C, lack the HDL_2_ fraction and that their HDL is depleted of cholesteryl esters [29]. These findings can be explained, in part, by reduced LCAT activity identified in FCS patients. Future studies will investigate mechanisms that link an *APOC2* or *LPL* genetic defect with the expression and/or regulation of activity of LCAT. Yet, our work suggests that increasing LCAT expression and/or activity might be a useful strategy to normalize cholesterol balance in plasma of patients with FCS. Whether this strategy would also apply to other cases of hypertriglyceridemia is unclear. Although hypertriglyceridemia is generally associated with decreased cholesterol esterification and accelerated HDL catabolism [16, 30], we found that the FC/CE ratio was not affected by the reduction of plasma TG levels in *apoc2* mutants fed an LFD (Fig 2). Similarly, the FC/CE ratio did not correlate with TG levels in human plasma (Fig 4D).

In summary, our data indicate that the FC/CE ratio is increased in both *apoc2* knockout zebrafish and LPL-deficient FCS patients. The increased FC/CE ratio may be triggered by insufficient plasma FC esterification, as liver *lcat* expression and plasma LCAT activity are reduced in *apoc2* mutant zebrafish and in human FCS patients, respectively. The biological significance of the distorted FC/CE ratio in FCS patients requires further studies.
